# Skin grafting: A simple solution to practice the technique and improve skills in young trainees

**DOI:** 10.4103/0970-0358.53028

**Published:** 2009

**Authors:** G. I. Nambi, Jeeth Jacob, Ashish Kumar Gupta

**Affiliations:** Department of Accident and Emergency, Apollo Hospitals, Graeme's Lane, Off Graeme's Road, Chennai, India; 2 Department of Plastic and Reconstructive Surgery, Christian Medical College and Hospital, Vellore, Tamil Nadu, India

**Figure 1:**
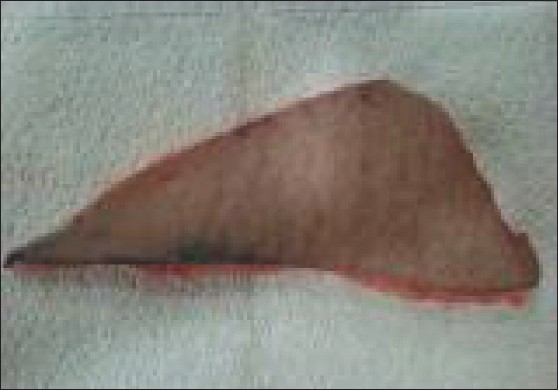
The specimen

**Figure 2:**
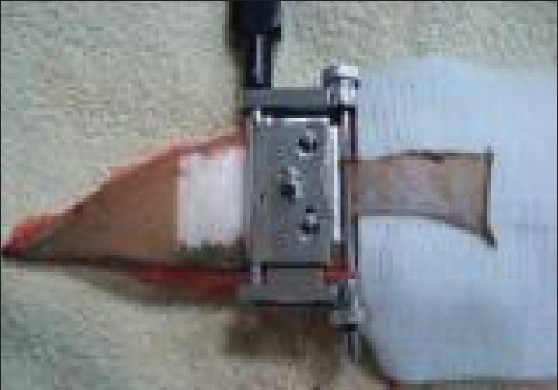
Split skin graft harvested with silver knife

Sir,

Skin grafting is a basic procedure in plastic surgery. However, young doctors may hardly get a chance to harvest a skin graft from an actual patient in the early days of their career. Yet, they need to practice the technique to master their skin graft harvesting skills. For this, several solutions have been described in the literature[1,2] involving synthetic materials. We found a simple way to practice the skill using abdominoplasty specimens [Figure 1] which have already been described for practising local flaps.[3] Depending upon the size of the specimen a Humby's or a Silver knife can be used. Traction is applied to one end of the specimen using a wooden board by an assistant and the operator himself gives traction to the other end with his left hand, just as is done in actual surgery. The size of the graft thus harvested varies depending upon the length and width of the specimen [Figure 2]. Abnormally thick specimens may have excessive convexity of their surface which can be corrected by trimming the excessive fat before practising the technique. By using the natural skin from abdominoplasty specimens and practising the actual technique, we found this to be enormously good for confidence-building in young trainees.
